# Genetics, Epigenetics, and the Environment: Are Precision Medicine, Provider Compassion, and Social Justice Effective Public Health Measures to Mitigate Disease Risk and Severity?

**DOI:** 10.3390/ijerph21111522

**Published:** 2024-11-16

**Authors:** Philip M. Iannaccone, Rebecca J. Ryznar, Lon J. Van Winkle

**Affiliations:** 1 Departments of Pathology and Pediatrics, Northwestern University, Evanston, IL 60611, USA; pmi@northwestern.edu; 2 Department of Biomedical Sciences, Rocky Vista University, Englewood, CO 80112, USA; rryznar@rvu.edu; 3 Department of Medical Humanities, Rocky Vista University, Englewood, CO 80112, USA; 4 Department of Biochemistry, Midwestern University, Downers Grove, IL 60515, USA

**Keywords:** public health, precision medicine, gene polymorphisms, toxic exposure risk, bias mitigation, social determinants of health, epigenetics of disease, adverse childhood experiences (ACEs), provider compassion, social justice

## Abstract

Environmental forces impacting public health include exposure to toxic substances, adverse childhood experiences (ACEs), diet, and exercise. Here, we examine the first two of these forces in some detail since they may be amenable to correction through cultural, medical, and practitioner intervention. At the same time, changing people’s dietary and exercise routines are likely more resistant to these interventions and are referred to only incidentally in this review. That is, societal efforts could prevent exposure to toxicants and ACEs—not necessarily requiring cooperation by the affected individuals—whereas changing diet and exercise practices requires an individual’s discipline. Toxic substances considered in this review include endocrine disruptors, arsenics, 2,3,7,8-Tetrachlorodibenzo-p-dioxin (TCDD), the organic solvent, Trichloroethylene (TCE), and the Benzo[*a*]pyrene (B[*a*]P) produced from incomplete combustion of tobacco and other organic materials. Exposure to each of these toxic substances may have serious adverse health effects, especially in genetically more susceptible individuals. For example, children of mothers exposed to the endocrine disruptor, Atrazine, have significantly lower birth length, weight, and head circumference. Moreover, male offspring exhibit genital abnormalities, and all of these effects may be transgenerational. However, analyses of interactions among genes, the environment, and epigenetic modifications have already revealed distinctive individual risks of adverse reactions to toxic exposure. So, interventions through precision medicine might improve the health of those exposed individuals. Adults previously exposed to more than one ACE (e.g., child abuse and inter-parental violence) are more likely to develop anxiety, cancer, and diabetes. Detecting ACE exposures in children in the general population is fraught with difficulty. Thus, the risks of ACEs to our health remain even more insidious than exposures to toxicants. Nevertheless, higher provider compassion is associated with significantly better clinical outcomes for patients with these afflictions. For all these reasons, the first major aim of this review is to recount several of the major forces contributing to or impairing public health. Our second major aim is to examine mitigating influences on these forces, including social justice and provider compassion in the setting of precision medicine. Idealistically, these mitigators might eventually lead to the development of more cooperative and compassionate cultures and societies.

## 1. Introduction

Health outcomes are influenced by social conditions, environment, and socio-economic position. But what can be made to mitigate the adverse effects of these factors? An old mariner’s adage, “You can’t navigate from lost”, applies here. As we navigate issues of public health, the environment, environmental justice, and health outcomes in the new era of personalized medicine, it serves us well to be aware of where we are [1,2] aided here by two major aims.

Aim 1. To recount several of the major forces contributing to or impairing public health (see Section 2, Section 3 and Section 4).

These forces include exposure to toxic substances, adverse childhood experiences (ACEs), diet, and exercise. Diet and exercise can be resistant to change even when using individualized interventions resembling precision medicine because they also require patients to make changes. Hence, although diet and exercise are impactful—for example, neighborhood walkability can cut the time that residents are sedentary and, thus, reduce their risks of obesity [3]—they will not be considered in detail here. 

Much more frequently, successful effort has been invested in reducing toxicant and ACE exposures, and more work is needed. We will consider here the harmful effects of 2,3,7,8-Tetrachlorodibenzo-p-dioxin (TCDD), Trichloroethylene (TCE), Benzo[*a*]pyrene (B[*a*]P), arsenics, and endocrine disruptors [4,5,6,7,8,9]. The effects of endocrine disruptors seem especially insidious. For example, women of reproductive age who are exposed to the endocrine disruptor, Atrazine, give birth to smaller children with reduced head circumferences, and their male offspring have genital abnormalities [10]. More concerning, these effects are likely transgenerational [11], and marginalized and socioeconomically disadvantaged persons have increased risks of exposure [12]. Even within groups of people, however, different individuals may have different risks from toxicant exposures because each person may have unique sets of genes and epigenetic modifications [13]. Hence, individualized treatments, including precision medicine, might be used to help them avoid toxicant exposure or prevent harm if they are exposed.

In addition, contact with toxic substances can lead to the development of adult diseases and disorders and may have an impact on their descendants [14]. Similarly, such diseases and disorders are more common among adults previously experiencing adverse childhood events, such as sexual abuse, regardless of toxicant exposure [15]. And the contributions of ACEs themselves as environmental causes of adult diseases and disorders are likely even more intractable than exposure to toxic substances (see Section 4 below). Moreover, adults exposed to more than one ACE (e.g., child abuse and inter-parental violence) develop health-threatening behaviors, such as drug abuse, as well as anxiety, cancer, and diabetes more often than their unexposed counterparts [15]. Detecting ACE exposures in children in the general population is, however, fraught with difficulty (see Section 4.1 below). Thus, the risks of ACEs to our health remain much less conspicuous than exposures to toxicants.

Aim 2. To examine mitigators of the forces that hinder public health.

Several mitigating influences on the forces outlined under Aim 1 above can foster public health. These mitigating effectors include provider compassion, precision medicine, and social justice. Because of the potential influences of these mitigators, we could become more cooperative and compassionate people and societies. Our commitment to social justice in the US and elsewhere is, however, only slowly emerging. For example, in Section 5 below, we discuss our response to the COVID-19 pandemic in the US as a model of societal compassion shortcomings [12]. And provider biases against some categories of patients, or even some patients on an individual level, lead to less than the best possible physical and mental medical outcomes [11,16,17,18,19]. But providers can be trained to become more empathetic and compassionate (e.g., [20,21,22,23]). And this training and the resultant behavior toward their patients also benefits practitioners by increasing the satisfaction they achieve from their work while decreasing the likelihood of them experiencing burnout. Moreover, such providers are increasingly more likely to advocate for social justice in medicine [24], which is known to have improved public health globally over the past 60 years [25]. Hence, a major goal of this review is to foster a continuing pursuit of social justice in medical practice.

More recently, we expanded our definition of precision medicine further to foster public health. Here, we define precision medicine as the ability (a) to detect genetic and epigenetic variations in individual susceptibilities to various toxicants and other vulnerabilities and (b) to protect and treat individuals based on their unique sensitivities. A comprehensive discussion of precision medicine can be found in reference [26]. And here, we expand this definition to include not only genetic and epigenetic information but also other unique characteristics of patients’ stories. Opportunities to utilize precision medicine are interspersed in the text of Section 2 and Section 3. Instances of the societal compassion needed to foster social justice are also interspersed in Section 2 and Section 3—and this compassion is considered in Section 5 as well—as are the potential impacts of provider compassion on individual patient’s health and wellbeing. 

## 2. Toxic Exposure, Disease, and Their Possible Solutions

### 2.1. Genetic Susceptibility

Drug metabolizing enzymes and transporter proteins vary in potency and effect. They detoxify by modification and removal of toxic substances. In the course of metabolism, genotoxic intermediates are produced that can cause mutations. Some are in critical genes and lead to severe disease outcomes. Moreover, toxic exposures can lead to epigenetic changes that contribute to poor outcomes, and these outcomes can be transgenerational without further toxic exposure [27]. Such metabolizing enzymes include the cytochrome P450 proteins. Detoxification can involve conjugation catalyzed by glutathione S transferase (GST), resulting in water solubility [28]. The hidden phenotypic variability among individuals can be established with high throughput genome sequencing and association studies that can identify individuals at risk for adverse outcomes. These data can be layered on exposure risk data to create an individual profile that can be used to intervene with emerging surveillance and treatment techniques [29]. While polygenic risk scores have also been developed and could become very desirable for individuals, so far, they appear to be more useful for groups of people than for individuals [30].

Genome-wide association studies [31] expand the knowledge base of polymorphic response to toxic exposures, and these expand the risk profiles available. Importantly, the sequence data include regulatory sequences such as enhancers, promoter areas, intronic controllers, and the important aspects of noncoding regions [32,33]. A combination of single-nucleotide polymorphisms (SNPs), copy number variations (CNV), prevalence of haplotypes, and combined polymorphisms has been used to establish risk profiles [29,34,35].

Total lifetime environmental exposures have come to be known as the exposome. The health outcomes in an individual are the result of the summed risk of probability of genotoxic metabolites from high exposure risk, adverse polymorphisms, and the production of mutations with health consequences. In principle, these can all be determined.

### 2.2. Available Data Concerning Toxic Exposures

Data relevant to toxic exposure is a robust part of cheminformatics. For example, structure-activity relationships are publicly available in databases of relevant exposure, including USDA-PDP and Toxcast 320. Additionally, data on toxic releases and reporting releases are available (Toxic Release Inventory [https://www.epa.gov/toxics-release-inventory-tri-program/what-toxics-release-inventory], URL accessed on 1 November 2024).

These exposure data can be expected to greatly increase in the near term. Increasing availability of sensors for air pollution creates very large data sets [36,37,38]. Analytical techniques are available and constantly improving for analyzing these large data sets and reducing them to practice (e.g., unsupervised learning, like clustering, and supervised/predictive learning). Indeed, this process can be expected to significantly accelerate as artificial intelligence (AI) is brought to bear. The rapid advances of AI methods and their availability all but ensure their application in these analyses [39].

### 2.3. Citizen Science

The concept of citizen science is to have a large number of interested and motivated individuals work on aspects of a scientific problem where knowledge, understanding, and direction come from a small number of professional scientists. Thus, data collection, observation, and other labor-intensive tasks related to a project are performed by large numbers of people in the general population interested in participating.

An example in environmental science was the study of diesel exhaust in New York City’s neighborhoods. The exhaust contains microparticulates with known adverse health effects [40,41]. The goal was to understand the distribution of such pollution in various neighborhoods. This was achieved by having individuals carry air monitors and track their movements. The surprising results indicated extreme variation across very small areas. They concluded that isolating point sources of pollution remained an important goal based on these data [42,43]. It is now well established that microparticulate exposure shortens the human life span [44,45,46].

As monitoring devices become more sophisticated [36] and geopositioning is more accessible, an expansion of data collection can involve more citizen scientists. Ultimately, the data may provide compelling support for mediating socio-economic and demographic exposure (and risk) disparities. Individual exposure experience and geopositioning data—combined with sequence data—can develop a risk profile for a given pollution event in each individual, particularly when analyzed along with individual genetic data. The monitoring can be supported by the development of next-generation sensors [36], especially as wearables.

Nevertheless, it can be argued that citizen science programs may suffer from an institutionalized lack of diversity. For example, species field reporting efforts have been reported to be dominated by older men [47]. Similarly, inequalities persist among those who choose to connect with ocean environments, although community-building educational efforts seem to broaden the diversity of people engaging in pro-environmental behaviors through citizen science [48]. And recruiting Black, Indigenous, and People of Color to speak at conferences for STEM students seems to enhance these educational efforts [49]. However, it seems unlikely that education alone can mitigate unjust risks of exposure to toxic chemicals among communities where the wealth of families varies considerably.

### 2.4. Understanding Disparity and Environmental Justice

An episode in Chicago provides a look at how insensitivity to population demographics and personal concerns leads to injustice. An industrial smokestack in a minority neighborhood was demolished, but the effort was botched with a significant release of dust and dust-borne toxic substances (e.g., mercury, sulfur dioxide, nitrogen oxide, and other pollutants). The residents were not warned prior to the effort or of the release of dangerous toxicants after demolition. The construction, moreover, was designed to proceed to transit centers with attendant traffic and air pollution. The same events, if they would occur at all, would have been very different in affluent neighborhoods (https://news.wttw.com/2021/04/11/year-after-smokestack-implosion-coated-little-village-dust-environmental-justice-fight, URL accessed on 1 November 2024). Unfortunately, exposure influenced by socio-economic position is all too common. For example, wealthy families are better able to move to “cleaner” counties than those households with less income [50]. Given that there are broad disparities in environmental quality and that those disparities directly affect health outcomes, professionals who deliver care need to become active advocates for justice to mitigate these inequitable risks and concomitant diseases [24]. Examples of how teams of healthcare professional students help one another become aware of such inaction and implicit biases are shown in reflections by students in Appendix E of Schwartz et al. [20] and Appendices A and B of Horst and associates [21]. In the latter case, one student began a reflection by stating that in “*my last reflection, I investigated my anxiety for our volunteer project selection. How, I wanted to work in the hospital compared to the homeless shelter. I wrote about some reasons for these emotions but during this week’s group meeting, I think I pin-pointed where my anxiety (and biases) stem from*”.

Marginalized populations, such as homeless people, have both greater risks of exposure to toxicants and less access to healthcare, so they experience much greater disease morbidity and mortality [12]. Hence, a tremendous range of outcomes exists for cardiovascular, lung, and neurodegenerative diseases as well as cancers, also influenced by whether practitioners exhibit empathy and are compassionate (see Section 5 below). If we expand this compassion to our culture as well as individual caretakers, we may hopefully begin to eliminate unjust differences in individual outcomes. This new justice can also be facilitated in controlled studies using wearable sensors to determine personal toxicant exposures, sequencing pertinent genes to detect enzyme polymorphisms, and determining profiles of microbiomes.

### 2.5. Further Examples of Environmental Health Concerns 

Well-known examples of environmental causes of diseases and disorders guide the way to successfully collect individualized data in order to provide personalized medicine in response to the environment. These examples include various sources of environmental endocrine disruptors. Disruptors may imitate normal hormones, antagonize them, alter the synthesis or activity of their receptors, or disrupt their production [51]. 

Atrazine has been used to improve crop production, but it also likely increases steroid synthesis and further processing in vertebrates. It upregulates genes needed for steroid synthesis and increases the activities of enzymes that catalyze the production of estrogen from testosterone. Likely, in these ways, frogs are feminized by Atrazine and are caused to produce tadpole hermaphrodites when their water is contaminated by this substance [52]. Moreover, Atrazine causes abnormal reproductive tract development, impairs egg production, and alters spawning in fish. Since one of the enzymes whose activity is increased by Atrazine is essential for normal estrogen synthesis from testosterone, the increased activity of this enzyme (aromatase) results in higher endogenous levels of estrogen and lower levels of testosterone in vertebrates [53]. 

Humans also suffer from Atrazine exposure. Children of mothers exposed to Atrazine have significantly lower birth length, weight, and head circumference. In addition, male offspring exhibit genital abnormalities. Such Atrazine exposure in rats also causes epigenetic modifications that are transgenerational [8] (see also Section 3 below). Of further concern, Atrazine and its metabolites persisted in humans for at least three years after the use of this substance was banned [54]. 

Thus, maternal exposure to Atrazine likely causes multiple abnormalities during the pre- and postnatal development of their children and grandchildren [10]. As a result, a class action lawsuit led the manufacturer to stop producing Atrazine and to provide materials needed to remove it from the water supplied by municipalities. In related circumstances it might also be possible to determine a citizen’s risk level according to their unique polymorphisms and Atrazine exposure. This could help to obviate a need to look for resultant health effects owing to personal exposures. 

As another classic example of disease-causing environmental toxic substances, groundwater may contain well-known and interrelated toxic substances, arsenates and arsenites. And these “arsenics” are present in groundwater in New Hampshire at relatively high natural levels. Arsenic exposure causes cancer and a number of other diseases and disorders [4]. Importantly, arsenic is also a widespread but geographically heterogeneous contaminant, particularly in rice, where cross-reacting transporters concentrate arsenic, when present, in the plant [5]. By altering the level of expression of the GLI oncogenes, arsenic can cause cancer as well as influence normal development by changing gene activity downstream to GLIs [55]. GLIs are transcription factors that regulate signal transduction, such as in the Sonic Hedgehog/Ptch/Gli pathway [32,56]. GLIs regulate the expression of genes in the pathway, including other GLI genes, and, thus, foster their dysregulation. Owing to their involvement in numerous developmental events, dysregulation of the genes in this pathway by arsenic is particularly far-reaching and insidious. However, arsenic must be metabolized to have many of its effects, as well as to be detoxified and excreted. There is considerable heterozygosity in the genes encoding enzymes involved in this metabolism [57,58]. Hence, genetic analyses might reveal people’s individual health risks owing to arsenic exposure.

Another well-known environmental toxic substance is 2,3,7,8-Tetrachlorodibenzo-p-dioxin (TCDD). Numerous industrial processes produce TCDD as a contaminant. In a widely studied event in Seveso, Italy, a large amount of it was released in 1974 [59]. Agent Orange is also contaminated with TCDD, and this herbicide was used heavily in Laos and Vietnam during the war. Dioxin is very stable and, thus, persists in the ground of those countries even today [60]. 

Its strong induction of a mixed-function oxidase in the liver and elsewhere—which depends on cytochrome P450—seems to result from TCDD’s association with a receptor in the cytoplasm, which then shuttles it to the nucleus [9,61]. While TCDD is extremely toxic to many mammalian species, cultured cells can resist its toxic effects even after induction of P450 activity. Hence, it is conceivable that its toxic effects can be assuaged. 

However, TCDD influences the expression of many other genes. But polymorphisms in the affected genes make it likely that exposed persons vary in their TCDD responses. Therefore, it may be possible to determine individual risks of diseases and disorders when they are exposed to TCDD using DNA and RNA sequencing. 

An infamous environmental toxic substance is the organic solvent Trichloroethylene (TCE). Its use extends widely from dry cleaning to industry, and inhalation is the most common means by which humans are exposed [62]. TCE has been used liberally at military bases, including Camp Lejeune, where many exposed personnel eventually developed cancers and Parkinson’s Disease [63]. Causality was clearly established by comparison with comparable military camps without TCE contamination, where these outcomes were not observed. But individual differences likely exist in response to TCE exposure owing to polymorphisms of the transporters and enzymes involved in clearing TCE [64]. These differences might be exploited to assess the relative risks and possible interventions for individuals. 

Finally, humans use CYP enzymes to produce benzo[*a*]pyrene diol-epoxide (BPDE) from Benzo[*a*]pyrene (B[*a*]P). Incomplete combustion of tobacco and other organic material produces B[*a*]P. Thus, high levels of B[*a*]P are present in the environment, and its metabolism to BPDE and other potential DNA adducts renders it highly carcinogenic [65]. Consequently, an individual’s CYP activity and ability to repair DNA determine their relative risk of developing cancer because the genes encoding the pertinent enzymes are highly polymorphic [65]. Persons who are highly vulnerable to B[*a*]P should especially avoid exposure to it. 

Wetlands also contribute to toxicant mitigation through the detoxification of fertilizer and pesticide runoffs [66]. Thus, the maintenance of wetlands (water margins) is crucial not only to our ecology but also to our health. While there are many ways wetlands affect our health, the mechanisms of this impact are poorly understood. It is clear, though, that as wetlands are diminished, their ability to detoxify fertilizer and pesticide runoffs is lost, and the environment is further contaminated, especially near farms. Causes of wetland breakdown include climate change, pollution, eutrophication, land use change, and biodiversity loss. Wetland loss has serious economic implications. Worldwide, local fishing and shell-fishing industries are highly dependent on coastal wetland habitats. For all these reasons and more [66], advocating for wetland maintenance could have major consequences for global public health.

## 3. Epigenetics of Exposure to Toxic Substances

Exposure to toxic substances is associated with a multitude of negative health outcomes. Numerous experimental and epidemiological investigations designed to study the effects of toxic exposure in humans bolster the Developmental Origins of Health and Disease hypothesis [67]. That is, exposure to toxic substances during development leads to epigenetic changes producing diseases and disorders in adults, although the precise molecular mechanisms of this causal sequence are not completely understood. Epigenetic modifications, including alterations in DNA methylation, histone modifications, and noncoding RNA expression, have emerged as potential mediators of the long-term health impacts of toxic exposure [68]. Therefore, unraveling epigenetic changes from toxic exposures that contribute to adverse health effects is important for safeguarding public health. An important caveat in the use of such epigenetic and genetic data, however, is that pertinent sociodemographic characteristics of the people studied are often poorly reported [69]. Hence, they may not apply especially to all genders/sexes, ages, and racialized groups of people.

Epigenetic alterations denote enduring shifts in gene expression that persist beyond the initial trigger, independent of changes in gene sequence or structure. These modifications encompass histone modification, DNA methylation, and noncoding RNA (ncRNA) activity. The epigenome encapsulates the entirety of these modifications within a cell at a given moment, while the genome encompasses the cell’s complete genetic material in the form of nucleotide composition. Histone modifications entail the addition or removal of specific chemical groups to these proteins, which serve as the structural units around which DNA is wound. These modifications—including acetylation, methylation, phosphorylation, ubiquitination, and sumoylation—form the histone code, orchestrated by various enzymes like histone acetyl transferases (HATs), histone deacetylases (HDACs), histone methyl transferases, and histone demethylases. Such modifications determine the expression status of a particular gene through changes in chromatin structure. Similarly, DNA can undergo methylation, primarily at CpG dinucleotides, regulated by DNA methyltransferases (DNMTs) and ten–eleven translocation methyl cytosine dioxygenase (TET) enzymes. Noncoding RNAs (ncRNAs), categorized into long noncoding RNAs (lncRNAs) and short ncRNAs (sncRNAs), exert regulatory control over gene expression. While lncRNAs influence gene expression through various mechanisms, sncRNAs predominantly modulate posttranscriptional gene expression. These epigenetic processes synergize to form intricate regulatory networks, which are crucial for development, phenotypic plasticity, and homeostasis maintenance [70].

For example, epigenetic reprogramming occurs during early development. Initially, post-fertilization, global reprogramming resets the zygote genome through DNA demethylation, histone modification, and ncRNA expression, enabling cell dedifferentiation. Subsequently, guided by DNA methylation, histone modification, and ncRNA activity, epigenetic programming directs embryogenesis and adult maturation. In addition, reprogramming also occurs in primordial germ cells (PGCs), which give rise to gametes. Here, imprinted genes undergo resetting by removal and re-establishment of the epigenetic marks that direct gene expression from the proper maternal vs. paternal alleles. PGCs experience genome-wide demethylation, histone modification changes, and altered ncRNA expression based on the embryo’s sex, ensuring accurate transmission of epigenetic information across generations [71]. Epigenetic mechanisms also operate to silence the genes on one of the two x chromosomes in female mammals for gene dosage compensation.

Altogether, the epigenome represents a biochemical language that functions to communicate with the genome about the timing and extent of gene expression that is cell and environmental cue-dependent. 

Many toxic substances have been studied for their effects on the epigenome [70] and, even further, the intergenerational transmission of these effects [72]. Some that have reproducibly shown epigenetic changes in humans following exposure include PM (particulate matter in air pollution), EDCs (endocrine disrupting chemicals), benzene, arsenic, cadmium, and other metals. Exposure pre-conception, in utero, and during critical developmental windows in early life can disrupt epigenetic programming patterns, potentially contributing to disease development later in life [70]. At any point throughout life, exposure can lead to an increase in the likelihood of developing diseases, including but not limited to respiratory diseases, cancer, neurodegeneration, and other mental health disorders [73]. 

Numerous studies have established connections between exposure to particulate matter (PM) from air pollution and alterations in DNA methylation, with pregnant women and children being particularly vulnerable. Previous investigations have revealed that both short-term and long-term exposure to ambient air pollution correlate with changes in DNA methylation levels. Methylation changes within DNA repair genes such as ERCC1, ERCC6, OGG1, MGMT, and HMLH1 have been observed, impacting their expressions and subsequently affecting the repair mechanisms activated in response to genetic damage induced by air pollution, therefore increasing the risk of carcinogenesis [74]. Similarly, differential DNA methylation levels have been detected in *AHRR*, *COL5A1*, *TNS1*, and *LINC00886*, accompanied by corresponding changes in their expression levels [75]. Additionally, researchers have explored the effects of PM exposure on expression patterns of DNA methyltransferases (DNMTs) and ten–eleven translocation (TET) enzymes. While a single treatment with PM2.5 (fine particulate matter less than 2.5 microns in diameter) for 24 h did not affect DNMT expression, it resulted in a significant decrease in TET expression. However, administering low concentrations of PM2.5 daily for 7 days increased the expression of TET1, TET2, and TET3, indicating differential effects based on acute or chronic exposure and highlighting the need for further investigation [75]. Altogether, these results suggest that exposure to particulate matter (PM) from air pollution induces alterations in DNA methylation and expression levels of epigenetic modifying enzymes, contributing to air pollution-related carcinogenesis through impacts on DNA repair genes’ expression and repair mechanisms.

Endocrine-disrupting chemicals (EDCs) comprise another type of environmental toxicant that has shown detrimental effects on the human epigenome. EDCs encompass various substances like medications, pesticides, plasticizers, and flame retardants. One recent review identified 37 studies linking adverse outcomes of EDC exposure with specific epigenetic changes, primarily DNA methylation and miRNA alterations, due to EDC exposure during different developmental stages [6]. Only 8 of these 37 studies delved into mechanistic investigations, particularly involving cigarette smoke and environmental chemicals like benzopyrene, benzophenone, and BPA polycyclic aryl hydrocarbons (PAH). Researchers suggest that EDCs not only influence the enzymes regulating epigenetic modifications but also affect levels of cofactors like S-adenosylmethionine (SAM) [75]. S-adenosylmethionine serves as the methyl donor in DNA cytosine methylation. Additionally, studies describe EDC effects on DNMT expression, either through receptors that directly regulate Dnmt mRNA expression or via an increase in miRNA-29 expression, leading to decreased Dnmt1, 3a, and 3b mRNA levels [76]. Furthermore, EDC exposure disrupts enzymes involved in DNA demethylation [77] and histone-modifying enzymes such as KAT2A and HDAC1-3 [78]. These results suggest that EDCs have the potential to modify the epigenome of those exposed both directly and indirectly. 

In addition to EDCs, another toxicant that many individuals are exposed to is benzene. Workers in various industries, such as those involved in petroleum refining, chemical manufacturing, and rubber production, are at risk of benzene exposure due to its presence in industrial processes. Additionally, benzene is a component of gasoline and is released into the air during fuel combustion, contributing to ambient air pollution. Individuals living near industrial facilities, gas stations, or heavy traffic areas may experience benzene exposure through inhalation of contaminated air. Moreover, benzene can leak into groundwater from underground storage tanks or spills or from release during fracking, potentially contaminating drinking water sources. Cigarette smoke is another significant source of benzene exposure, with tobacco smoke containing high levels of this hazardous chemical. One recent study reported that workers exposed to low doses of benzene show significant hypomethylation of long interspersed element 1 (LINE-1) and Alu DNA in leukocytes, along with gene-specific hypermethylation of cyclin-dependent kinase inhibitor 2B (*CDKN2B*) and hypomethylation of melanoma-associated antigen 1 (*MAGE1*) [79]. These alterations in DNA methylation resemble the epigenetic patterns observed in malignant cells, suggesting a potential link between low-level benzene exposure, aberrant DNA methylation, and cancer. Longitudinal studies reveal a more pronounced decrease in DNA methylation over an 8-year period among exposed individuals compared to controls, further implicating benzene exposure in epigenetic modifications [80]. Also, analysis of plasma miRNAs in benzene-exposed workers revealed differential expression of miR-638, let-7f-5p, and miR-223-3p, suggesting their potential role in benzene-induced hematologic toxicity. Pathway analysis implicated focal adhesion as a key pathway affected by benzene exposure [81], and perturbation of this pathway has been implicated in many human pathologies [82]. Moreover, in a separate study, benzene exposure was associated with the upregulation of specific miRNAs, such as miR-154, miR-487a, miR-493-3p, and miR-668, which have been linked to leukemia [83]. In summary, benzene has the potential to cause exposure-induced epigenetic changes through dysregulation of DNA methylation patterns and changes in miRNAs. 

Along with PM, EDCs, and benzene, exposure to toxic metals can also perturb the epigenome. Plasma levels of 21 metals, along with the methylation status of over 600 genes, were assessed in metal-exposed (individuals residing near a metal mining area) and unexposed cohorts. Results showed methylation alterations in *NFKB1*, *CDKN2A*, *IGF2*, and *ESR1* genes among individuals with prolonged human exposure to metals [84]. Changes in methylation patterns of these genes suggested disruption of immune response and oncogenesis through the dysregulation of PI3K, MAPK, and NF-κB pathways [84]. Many other studies have shown evidence to suggest that heavy metal exposure could contribute to induced epigenetic changes that led to cancer [85]. For example, cadmium, a toxic metal widely present in the environment, has been extensively studied for its impact on the human epigenome. Exposure to cadmium primarily occurs through various routes, including the consumption of plant-based foods, certain types of seafood, tobacco smoking, and industrial emissions. Research indicates that cadmium can modify epigenetic patterns in placental, fetal, and newborn DNA, with some studies noting significant sex-specific differences in cadmium-related DNA methylation alterations. Poor fetal growth, teratogenic effects, and preeclampsia have been documented in association with cadmium exposure. These poor health outcomes associated with cadmium exposure are linked to DNA methylation changes and interference with the activity of de novo DNA methyltransferases critical for development [86]. 

As discussed above, arsenic is yet another pervasive environmental toxicant affecting millions of individuals worldwide, and it can cause arsenicosis (arsenic poisoning) in chronically exposed individuals. One study identified a comprehensive interactome of hypermethylated genes enriched for their involvement in arsenic-associated diseases, including cancer, heart disease, and diabetes [87]. These researchers concluded that they had discovered an arsenic-induced tumor suppressorome comprising 17 tumor suppressor genes commonly silenced in human cancers [87]. Identifying interactomes for other toxicants would provide insight into underlying molecular mechanisms governing the diseases they induce (https://geiselmed.dartmouth.edu/childrenshealth/, URL accessed on 1 November 2024)

By understanding how toxin-induced epigenetic changes contribute to human disease, this information can be used to influence public health policies to help affected communities. Research should focus on determining not only specific tissues targeted following toxic exposure but also evaluating the specificity of epigenetic changes and how these changes can directly or indirectly lead to pathology in humans. 

## 4. Adverse Childhood Experiences Contribute to Environmental Causes of Adult Diseases That May Be Even More Intractable than Exposure to Toxic Substances

Adverse childhood experiences also contribute to environmental causes of adult diseases, and the epigenetics of these environmental effects need to be elucidated [88]. Especially in developed countries, adverse childhood experiences (ACEs) are associated with health-threatening behaviors, such as smoking, later in life. For these and other reasons, adults who experienced even one ACE, like physical abuse as a child, have an increased risk of depression and cardiac and respiratory diseases. Furthermore, adults exposed to more than one ACE (e.g., child abuse and inter-parental violence) are more likely to develop the latter conditions, plus anxiety, cancer, and diabetes [15]. Moreover, numerous epigenetic changes likely mediate the development of these psychosocial conditions. And direct evidence is emerging for epigenetic modifications associated with the occurrence of adult metabolic diseases owing to ACEs [88]. Perhaps not surprisingly, such epigenetic changes are also transgenerational [89,90]. Unfortunately, more than 60% of children in the US [91,92] and worldwide [93,94] experience at least one ACE, and it is more difficult to screen for ACEs than other environmental conditions, such as air pollution. Consequently, we argue that the health threat of the ACE component of the environment may be even more resistant to solution than prevention of environmental toxic exposure.

### 4.1. Childhood Abuse, Household Dysfunction, and Adult Diseases

Not only do over half of the children in North America and Europe experience abuse and neglect, but these experiences lead to both serious physical and mental disorders over the victims’ lifetimes and a high financial burden to care for them [95]. Though not defined in identical ways by all investigators, lists of ACEs are published (e.g., [96]) and include parental incarceration, drug abuse, mental illness, and separation; domestic violence; physical and emotional neglect; and emotional, physical, and sexual abuse of victims [97]. Over the course of their lives, these victims are more likely than ACE-free persons to smoke, abuse drugs and alcohol, experience anxiety and depression, and develop obesity, diabetes, cancer, cardiovascular disease, and respiratory illnesses [15]. And the economic impact of these scenarios is immense. For the six types of poor health and their risk factors associated with ACE, the annual cost to care for affected individuals in Europe and North America was estimated to be $1.3 trillion in 2017 [15]. An Adverse Childhood Experiences Scale [98] has been used to identify parents who experienced such adversity as children for further study of the impact of their experiences on their families (e.g., [99]). But of course, such scales lack sensitivity as tests for identifying ACEs in the general population.

### 4.2. Screening for Adverse Childhood Experiences and Their Possible Treatment or Prevention

While steps to intervene and treat or prevent the consequences of ACEs can be successful [100], ACE training remains rare among healthcare professional students [15]. And adequate screening for child abuse and neglect is only now evolving [101]. The ability to reliably identify ACEs in the general population should foster efforts to add this knowledge and its use to the curricula of healthcare providers and their students.

In this regard, the promising work of Hanson and associates [101] involves using existing electronic health records, natural language processing (NLP), and expert review to develop a lexicon of child neglect and abuse. They plan to improve their NLP algorithm using a more diverse and larger population sample. Also promising is the use of artificial intelligence to develop such lexicons, although, unfortunately, this use is lagging behind other medical applications [102]. In addition, provider awareness of the frequency of ACEs—and compassion for its victims—should help to alleviate resultant diseases and disorders. Providers must learn each patient’s unique story in order to employ the precision medicine techniques needed to mitigate their risk of disease and aid recovery from their disorders [12,103,104,105].

## 5. Greater Compassion Could Help to Mitigate Environmental Causes of Disease Risk and Severity

### 5.1. Response of the US Healthcare System to the COVID-19 Pandemic as a Model of Societal Compassion Shortcomings

In part III of her book, “*Legacy: A Black Physician Recons with Racism in Medicine*”*,* Uché Blackstock uses the COVID-19 pandemic to illustrate how deeply racism is embedded in every aspect of our lives in the US [12] and likely elsewhere. For example, in December 2020, 52-year-old Dr. Susan Moore—a black woman physician infected with COVID-19—proclaimed from her hospital death bed with an oxygen tube in her nose that her white doctor “made (her) feel like…a drug addict even though he knew she was a physician. He did not even listen to (her) lungs; he didn’t touch (her) in any way” [12] (pp. 236–237). He suggested only that she go home. She was dead less than three weeks later. Unfortunately, even in 2024, the words of Martin Luther King Jr. still ring true, “Of all forms of discrimination and inequalities, injustice in health is the most shocking and inhumane”, in part because of this lack of provider compassion.

Gilbert [106] defines compassion as “a sensitivity to suffering in self and others with a commitment to try to alleviate and prevent it.” And he argues for the creation of a compassionate world by fostering cooperation over competition [107]. While seeming, at first, highly idealistic, his sobering arguments apply to global public health, including improving dangers to ecology around the globe [108]. To date, however, many firms expend resources on waste management using the demographics of local populations [109,110]. Nevertheless, on a more personal level, higher healthcare provider empathy and compassion clearly contribute to the outcomes of precision medicine by improving the quality of patient care while decreasing the probability of practitioner burnout [16,17]. For example, practitioner empathy reduces the risk of serious complications in diabetic patients [18]. When such conditions become life-threatening, provider compassion improves not only the physical aspects of patients’ conditions. This compassion also decreases the rate at which these patients experience psychological distress, such as post-traumatic stress disorder [19]. Unfortunately, these compassionate measures were not offered to Dr. Moore by her doctor, nor do they usually extend to communities of color in the US more widely. 

Owing to difficulties in recognizing and measuring compassion in societies and cultures more broadly [111], we limit discussion of the benefits of empathy and compassion to that which is (1) perceived and recognized by patients and (2) self-reported mainly by nursing and medical students and practitioners [16,17,18]. We argue that such perception of provider compassion by patients and self-reported empathy by practitioners inform public health in order to mitigate disease risk and severity.

### 5.2. Provider Compassion, Precision Medicine, and Public Health 

In their comprehensive systematic review of empathy in healthcare, Nembhard and associates [16] included 455 articles and 470 studies. They found the predominant measures of empathy in these articles to be self-reported empathy in reliable and valid surveys, such as The Jefferson scale of Empathy (62% of studies) and patients’ perception of clinicians (29% of studies). These studies involved various aspects of empathy, such as predictors of empathy in practitioners. In the 42% of the studies examining the outcome of higher provider empathy, significantly better clinical outcomes, patient experiences and states, provider performances, and patient behavior/adherence were reported 81, 82, 84, and 100% of the time, respectively. And of the randomized control studies pertinent to the latter four types of results, 17 of 18 (94%) reported positive and significant effects of higher empathy [16]. Moreover, numerous methods are now available to foster provider compassion and prevent burnout [20,21,22,23]. Consequently, the impact of greater provider empathy and compassion—as a component of precision medicine—is significant. Again, this precision medicine and compassion must include learning each patient’s unique story [12,103,104,105]. Ultimately, this can lead to overall improvements in public health. Hence, our ability to increase healthcare practitioners’ empathy through training interventions ought to be exploited vigorously [16,17,18,23]. Beneficial epigenetic changes are also possible through supportive social environments (e.g., [112]).

## 6. Conclusions: What Else Can Be Undertaken?

In addition to critical reflection on service to the community to foster bias mitigation and compassionate behavior in providers and their students [20,21,22], this activity also promotes practitioner advocacy to mitigate the risks of toxic exposure, especially in more vulnerable and marginalized communities [24]. As cultures and societies, and as expressed by McKeachie [113], ultimately, we should “Teach (our) students to reflect both in and out of class. That reflection should never stop because conscious reflection on values is perhaps the cornerstone of the ethics of teaching.” Increased reflective capacity in prospective medical students helped them seek to communicate more effectively with patients, as shown in appendices C and D of Schwartz et al. [20]. And compassion, trust, and good relationships between patients and their healthcare providers are likely as important to effective treatment and prevention as the broader efforts discussed above [16]. Only through such reflection by most citizens can we better communicate with one another and, thus, produce and sustain equity and justice in healthcare and society more widely.

Diversity in healthcare professionals is also critical to recognizing the disparities that affect an individual’s health [12]. Minority groups are disproportionately underrepresented in most colleges of medicine and other healthcare disciplines (The Sullivan Commission (2004) “Missing Persons: Minorities in the Health Professions” https://campaignforaction.org/wp-content/uploads/2016/04/SullivanReport-Diversity-in-Healthcare-Workforce1.pdf, accessed on 1 August 2024; https://www.aamc.org/media/8816/download, accessed on 1 August 2024, [114]) [115]. And rectification of this disproportion would significantly improve public health, especially among marginalized persons [12]. For example, food insecurities contribute significantly to disparities in the incidences of chronic diseases [116], and greater participation of minority groups in the direct provision of medical care should help reduce these insecurities [12]. 

Beyond recognition of variation in response to environmentally induced injury, practical actions to eliminate environmental injustice (e.g., exposure risk by zip code) must be taken. Advocacy for intelligent clean-up of toxic sites (e.g., through Superfund program efforts) wherever they occur needs to continue. Effective new therapies will be required to leverage the increasingly high-resolution genetic, epigenetic, and exposure data for individuals into improved health outcomes. There remains an obligation to develop these approaches with regard to the ethics of data management and privacy.

With an “all hands on deck” approach, our society can achieve environmental and healthcare delivery justice to the benefit of the public health of all of us.

## Data Availability

All data are included in this paper.
